# Asymptomatic Intestinal Colonization with Protist *Blastocystis* Is Strongly Associated with Distinct Microbiome Ecological Patterns

**DOI:** 10.1128/mSystems.00007-18

**Published:** 2018-06-26

**Authors:** M. E. Nieves-Ramírez, O. Partida-Rodríguez, I. Laforest-Lapointe, L. A. Reynolds, E. M. Brown, A. Valdez-Salazar, P. Morán-Silva, L. Rojas-Velázquez, E. Morien, L. W. Parfrey, M. Jin, J. Walter, J. Torres, M. C. Arrieta, C. Ximénez-García, B. B. Finlay

**Affiliations:** a Laboratorio de Inmunología del Departamento de Medicina Experimental, UNAM, Mexico City, Mexico; b Michael Smith Laboratories, Department of Microbiology & Immunology, University of British Columbia, Vancouver, British Columbia, Canada; c Department of Physiology & Pharmacology, University of Calgary, Calgary, Alberta, Canada; d Department of Pediatrics, University of Calgary, Calgary, Alberta, Canada; e Department of Biochemistry and Microbiology, University of Victoria, Victoria, British Columbia, Canada; f Department of Zoology, University of British Columbia, Vancouver, British Columbia, Canada; g Department of Botany, University of British Columbia, Vancouver, British Columbia, Canada; h Department of Agricultural, Food and Nutritional Sciences, University of Alberta, Edmonton, Alberta, Canada; i School of Life Sciences, Northwestern Polytechnical University, Xi’an, China; j Unidad de Investigación en Enfermedades Infecciosas, UMAE Pediatria, IMSS, Mexico City, Mexico; k Department of Biochemistry and Molecular Biology, University of British Columbia, Vancouver, British Columbia, Canada; University of Colorado Denver

**Keywords:** *Blastocystis*, gut microbiome, eukaryome, host-microbe interactions, microbial ecology

## Abstract

*Blastocystis* is the most prevalent protist of the human intestine, colonizing approximately 20% of the North American population and up to 100% in some nonindustrialized settings. *Blastocystis* is associated with gastrointestinal and systemic disease but can also be an asymptomatic colonizer in large populations. While recent findings in humans have shown bacterial microbiota changes associated with this protist, it is unknown whether these occur due to the presence of *Blastocystis* or as a result of inflammation. To explore this, we evaluated the fecal bacterial and eukaryotic microbiota in 156 asymptomatic adult subjects from a rural population in Xoxocotla, Mexico. Colonization with *Blastocystis* was strongly associated with an increase in bacterial alpha diversity and broad changes in beta diversity and with more discrete changes to the microbial eukaryome. More than 230 operational taxonomic units (OTUs), including those of dominant species Prevotella copri and Ruminococcus bromii, were differentially abundant in *Blastocystis*-colonized individuals. Large functional changes accompanied these observations, with differential abundances of 202 (out of 266) predicted metabolic pathways (PICRUSt), as well as lower fecal concentrations of acetate, butyrate, and propionate in colonized individuals. Fecal calprotectin was markedly decreased in association with *Blastocystis* colonization, suggesting that this ecological shift induces subclinical immune consequences to the asymptomatic host. This work is the first to show a direct association between the presence of *Blastocystis* and shifts in the gut bacterial and eukaryotic microbiome in the absence of gastrointestinal disease or inflammation. These results prompt further investigation of the role *Blastocystis* and other eukaryotes play within the human microbiome.

**IMPORTANCE** Given the results of our study and other reports of the effects of the most common human gut protist on the diversity and composition of the bacterial microbiome, *Blastocystis* and, possibly, other gut protists should be studied as ecosystem engineers that drive community diversity and composition.

## Abstract (precis)

Given the results of our study and other reports of the effects of the most common human gut protist on the diversity and composition of the bacterial microbiome, *Blastocystis* and, possibly, other gut protists should be studied as ecosystem engineers that drive community diversity and composition.

## INTRODUCTION

*Blastocystis* is the most prevalent protozoan resident of the human intestine, colonizing approximately 0.5 to 30% of humans in industrialized countries and 30 to 100% in nonindustrialized societies (1–7). *Blastocystis* is transmitted via the oral-fecal route, and colonization has been linked to poor hygiene, animal contact, and contamination of food and water sources (4, 8). And yet, *Blastocystis* is also common in industrialized populations, with 20 to 50% prevalence among healthy cohorts in Europe (9, 10), and interestingly, is associated with high income in Denmark (11). Similarly, its role in human health and disease is controversial. *Blastocystis* has been associated with a variety of gastrointestinal symptoms (12–14) and inflammation, both within the gut (15) and systemically (16, 17). Several studies have suggested that *Blastocystis* may play a role in the development of irritable bowel syndrome (IBS) (11, 18, 19). However, *Blastocystis* has often been described as an asymptomatic member of the normal intestinal microbiota (7, 9, 10, 20) and has even been inversely associated with body mass index and Crohn’s disease (7). It remains unclear how *Blastocystis* is capable of acting as an opportunistic pathogen or a commensal or a beneficial microbe and to what extent this depends on the specific circumstances of its host.

The plethora of human-associated microbiome studies documenting the role of the microbiome in health and disease has directed considerable attention to the factors that influence microbiome diversity and composition. To date, diet (21, 22), antibiotics (23), age (24), inflammation (25), and to a lesser extent, host genetics (26–28) have been shown to impact the ecology of the gut bacterial community, yet most studies are limited to profiling bacteria and seldom consider interdomain, multitrophic interactions as one of these factors. The relationship between human-associated gut protists and the resident gut bacterial community has only recently begun to be explored. Pathogenic protists, such as *Giardia* and Entamoeba histolytica, are associated with an increase in gut bacterial diversity or with compositional differences (29–32), likely due to the mucosal damage and inflammation they cause in the intestinal mucosa. Interestingly, nonpathogenic protists may also cause major shifts in the bacterial microbiota: Morton et al. document an association between the presence of *Entamoeba* and increased diversity and compositional shifts in the bacterial microbiota in asymptomatic individuals, a portion of whom likely harbor nonpathogenic Entamoeba dispar (33). Recent reports in humans also suggest that *Blastocystis* is associated with compositional and diversity differences of the bacterial gut microbiota (2, 7, 34). However, given that this protist is known to cause disease in certain individuals and that the reported results are confounded by disease status, it remains unknown whether the changes in bacterial microbiomes are due to the presence of *Blastocystis* or to ongoing inflammation. Differentiating whether *Blastocystis* causes direct ecological effects on the bacterial microbiome or if microbiota shifts are mainly mediated through immune and physiologic changes associated with *Blastocystis*-associated inflammation will help determine the ecological consequences of decreased *Blastocystis* incidence in the westernized gut human microbiome.

In light of this, we aimed to study the gut microbiome in relation to *Blastocystis* colonization in a large sample of healthy individuals residing in a semi-industrialized setting in rural Mexico. Through this investigation, we detected marked taxonomic and functional bacterial differences associated with asymptomatic colonization of *Blastocystis* that support its role as a common eukaryotic gut commensal that drastically influences the bacterial microbiota through currently unknown mechanisms.

## RESULTS

### Prevalence of *Blastocystis.*

We studied 156 individuals from the town of Xoxocotla, in the state of Morelos, Mexico. This population was deemed free of gastrointestinal symptoms associated with disease, according to physical examination by a clinician and data obtained from the Rome III questionnaire (see Materials and Methods for criteria in this questionnaire). Besides the Rome III questionnaire, a complete medical history was carried out for each individual, emphasizing inflammatory symptoms and antibiotic use in the 6 months preceding the clinical assessment and sampling. None of the selected samples came from individuals that reported antibiotic use or previous inflammatory conditions. The gender distribution was 84 women (53.8%) and 72 men (46.2%), with a median age of 27 years (10 to 53 years). The majority of individuals in the study had some degree of education (84.6%). The most frequent level was secondary school (33.0%), followed by primary school (32%), preschool (26%), technical education (8.4%), and professional studies (0.49%) (see Table S1 in the supplemental material for baseline characteristics of study participants). The status of colonization by *Blastocystis* and other protozoa (Cryptosporidium parvum, Entamoeba histolytica*/*E. dispar, and Giardia intestinalis) was determined by microscopy and quantitative PCR (qPCR) assays targeting regions of the small subunit (SSU) rRNA gene to detect and quantify levels directly from human stool specimens. *Blastocystis* colonization was detected in 65% of individuals (102/156), 52% of whom (53/102) were women. There was no difference in the mean ages of *Blastocystis*-positive versus -negative individuals (27.8 ± 8.4 years [mean ± standard deviation] versus 28.1 ± 7.9 years, respectively; Student’s *t* test, *P* = 0.83). Targeted 18S rRNA gene sequencing results allowed further resolution of *Blastocystis* taxonomy, showing that *Blastocystis* subtype 3 (ST3) is the prevalent subtype colonizing this human population. We did not identify any individuals infected by Crysptosporidium parvum or Giardia intestinalis but did observe coinfection with Entamoeba histolytica/E. dispar in five individuals, who were excluded from the bioinformatics analysis in order to assess the effect of *Blastocystis* only.

10.1128/mSystems.00007-18.4 TABLE S1  Characteristics of study population. Download TABLE S1, DOCX file, 0.04 MB. Copyright © 2018 Nieves-Ramírez et al. 2018 Nieves-Ramírez et al. This content is distributed under the terms of the Creative Commons Attribution 4.0 International license.

### Intestinal microbiota composition and *Blastocystis* infection. (i) Prokaryotes.

**FIG 1:**
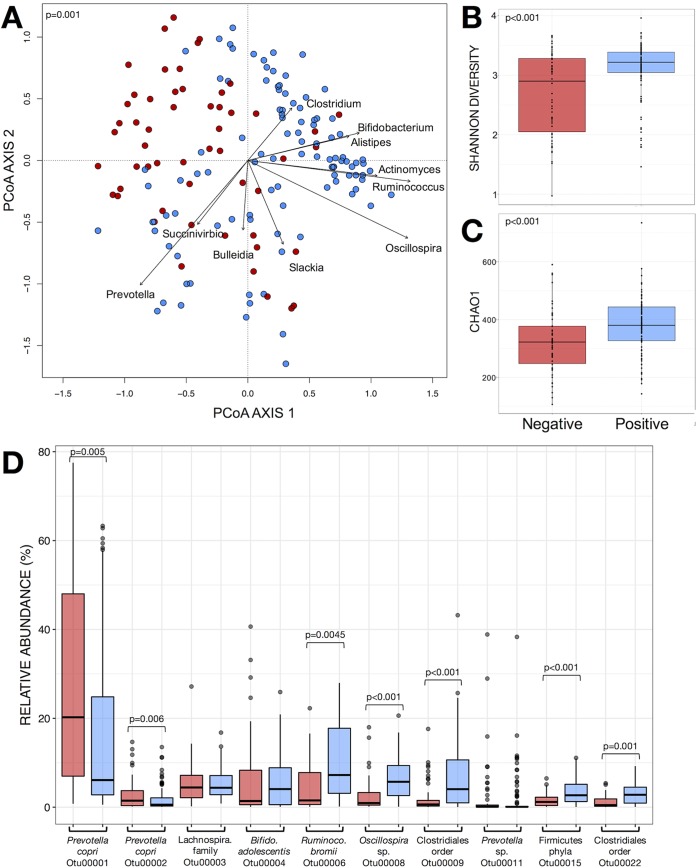
Variations in beta and alpha diversity of gut microbiome bacterial communities in relation to presence of *Blastocystis*. (A) Principal-component analysis (PCoA) ordination of variation in beta diversity of human gut bacterial communities based on Bray-Curtis dissimilarities among fecal samples. Colors represent the presence of *Blastocystis* in gut microbial communities (red for negative and blue for positive), and arrows represent the significant (*P* < 0.001) correlations between PCoA axes versus the relative abundances of bacterial genera in communities. (B and C) Shannon diversity (B) and Chao1 estimated richness (C) display differences in alpha diversity. Significant differences are shown by *P* values of Mann-Whitney tests for comparison between 2 groups. (D) Relative abundances of the 10 most abundant bacterial OTUs relative to the presence of *Blastocystis*. Significant differences are shown, calculated by DESeq2 (Wald test plus FDR).

We determined the bacterial community by amplification and sequencing of the 16S rRNA gene (V3 region). *Blastocystis* colonization was associated with profound changes in bacterial alpha and beta diversity (Fig. 1) (for analysis of alpha diversity, Chao1 and Shannon indices and Mann-Whitney test were used [*P* < 0.001], and for analysis of beta diversity, principal-component analysis [PCoA] and permutational multivariate analysis of variance [PERMANOVA] were used [*P* < 0.001]). The marked change in beta diversity can be explained by large differences in the abundances of predominant bacterial taxa, including Prevotella copri, Prevotella stercorea, Ruminoccoccus bromii, Alistipes putredinis, *Bacteroides* species, Bifidobacterium longum, and *Oscillospira* species (DESeq2, Wald test, and false discovery rate [FDR], *P* < 0.05) (Table 1; the full list of differential operational taxonomic units [OTUs] is in Table S2). The presence of *Blastocystis* was associated with significant differences in 7 of the 10 most abundant OTUs, including Prevotella copri and Ruminococcus bromii, the two most abundant taxa in the data set (Fig. 1D; Table 1). Similarly, at the genus level, 7 of the 10 most abundant genera, comprising ~65% of all bacterial sequences, were significantly different in relation to their abundances in the bacterial community associated with *Blastocystis* colonization (Fig. S1).

10.1128/mSystems.00007-18.1 FIG S1  Relative abundances of the 10 most abundant bacterial genera relative to the presence of *Blastocystis*. Significant differences are shown, calculated by DESeq2 (Wald test plus FDR). Download FIG S1, TIF file, 2.3 MB. Copyright © 2018 Nieves-Ramírez et al. 2018 Nieves-Ramírez et al. This content is distributed under the terms of the Creative Commons Attribution 4.0 International license.

10.1128/mSystems.00007-18.5 TABLE S2  Differential abundances of microbial OTUs (FDR < 0.05) between colonized and noncolonized individuals, calculated by DESeq2. Download TABLE S2, XLSX file, 0.1 MB. Copyright © 2018 Nieves-Ramírez et al. 2018 Nieves-Ramírez et al. This content is distributed under the terms of the Creative Commons Attribution 4.0 International license.

10.1128/mSystems.00007-18.6 TABLE S3  Differential PICRUSt-predicted metabolic pathways between *Blastocystis*-positive and -negative individuals. Download TABLE S3, XLSX file, 0.03 MB. Copyright © 2018 Nieves-Ramírez et al. 2018 Nieves-Ramírez et al. This content is distributed under the terms of the Creative Commons Attribution 4.0 International license.

**TABLE 1:** Differential abundances of taxa in relation to *Blastocystis* colonization calculated by DESeq2^a^

OTU_taxon	DESeq2 value for:					
	baseMean	log_2_ fold change	lfcSE	stat	*P* value	padj
*Bacteria*						
Otu00001_*Prevotella*_*copri*	58,635.572	−1.063	0.275	−3.863	1.12E−04	5.39E−03
Otu00002_*Prevotella*_*copri*	6,577.601	−1.179	0.309	−3.818	1.34E−04	6.41E−03
Otu00006_*Ruminococcus*_*bromii*	13,423.877	1.768	0.358	8.463	3.42E−04	4.50E−03
Otu00008_*Oscillospira*_sp.	2,952.294	0.983	0.138	7.133	9.79E−13	2.41E−10
Otu00009_*Bacteria*	3,050.404	1.348	0.217	6.211	5.26E−10	7.28E−08
Otu00013_*Prevotella*_*stercorea*	3,848.363	−1.614	0.358	−4.508	6.53E−06	4.30E−04
Otu00014_*Clostridiales*	1,022.155	0.707	0.142	4.987	6.12E−07	4.91E−05
Otu00015_*Firmicutes*	2,174.964	2.464	0.365	−6.741	1.57E−11	3.00E−09
Otu00018_*Alistipes*_*putredinis*	927.977	0.970	0.173	5.617	1.95E−08	2.18E−06
Otu00019_*Bifidobacterium*_*longum*	3,202.069	−2.947	0.242	−12.156	5.35E−34	5.92E−30
Otu00022_*Clostridiales*	1,449.441	0.890	0.153	5.802	6.55E−09	7.71E−07
Otu00025_*Oscillospira*_NA	1,524.800	−2.683	0.254	−10.575	3.91E−26	8.74E−23
Otu00030_*Bacteria*	2,201.012	−1.727	0.268	−6.446	1.15E−10	1.79E−08
Otu00032_*Barnesiellaceae*	499.952	−1.970	0.247	−7.964	1.67E−15	5.77E−13
Otu00041_*Rikenellaceae*	382.230	1.081	0.228	4.745	2.09E−06	1.51E−04
Otu00043_*Clostridiales*	482.388	1.111	0.209	5.313	1.08E−07	1.04E−05

Eukaryota						
OTU_8514_*Blastocystis*_sp._subtype_3	40.584	5.362	0.503	10.656	1.64E−26	5.80E−24
OTU_8487_*Blastocystis*_sp._subtype_3	36.126	5.202	0.498	10.444	1.56E−25	2.75E−23
OTU_5808_*Hymenolepis*_*nana*	42.245	−3.377	0.557	−6.066	1.31E−09	3.56E−08
OTU_5817_*Hymenolepis*_*nana*	37.953	−3.581	0.545	−6.574	4.91E−11	1.73E−09

a Results from the 30 most abundant taxa in the data sets are included. For a complete list, see Table S2. baseMean, mean of normalized counts; lfcSE, standard error of log fold change; stat, Wald statistic; padj, adjusted *P* value.

Given the metadata collected in this cohort (age, education level, and family relationships), we captured the effects of the *Blastocystis* colonization on the gut bacterial microbiome while controlling for potential confounding variables using MaAsLin (35). MaAsLin is a multivariate linear modeling tool with boosting that tests for associations between specific microbial taxa and continuous and/or Boolean metadata. This method reduces the total amount of correlations to be tested, therefore allowing for improvements in the speed and the robustness of the additive general linear models. With MaAsLin, we found a significant association between most of the differential taxa (previously obtained through the DESeq2 analysis) and *Blastocystis* status, whereas no other variable explained the taxonomic differences observed in colonized individuals (Table S4).

10.1128/mSystems.00007-18.7 TABLE S4  Bacterial taxa associated with *Blastocystis* colonization corrected for possible confounding effects using MaAsLin. Direction of the correlation coefficient is in relation to the data for the noncolonized group. Findings are FDR corrected. Download TABLE S4, DOCX file, 0.1 MB. Copyright © 2018 Nieves-Ramírez et al. 2018 Nieves-Ramírez et al. This content is distributed under the terms of the Creative Commons Attribution 4.0 International license.

We also controlled for the effect of sequencing depth influencing our results, as previously described by Weiss et al. (36), and found that while sequencing depth did impact the ordination of our data, the influence of *Blastocystis* species described in our study is not driven by the variation in sequencing depth across samples (Fig. S3A) (PERMANOVA, *P* = 0.5).

### (ii) Eukaryotes.

We determined the eukaryotic microbiota by amplification and sequencing of the 18S rRNA gene (V4 region). In the 18S rRNA gene data set, 63 samples had fewer than 1,000 sequences per sample after applying filtering steps (see Materials and Methods), which prompted their removal from the data set. Thus, 93 samples (*N*_pos_ = 60 and *N*_neg_ = 33, from colonized and noncolonized individuals, respectively) were retained for ecological analysis of this data set. There were no significant differences with respect to baseline characteristics between the reduced number of samples and the original data set used for 16S rRNA gene analysis (Table S5).

10.1128/mSystems.00007-18.8 TABLE S5  Baseline characteristics in reduced cohort used in 18S sequencing analysis. Download TABLE S5, DOCX file, 0.04 MB. Copyright © 2018 Nieves-Ramírez et al. 2018 Nieves-Ramírez et al. This content is distributed under the terms of the Creative Commons Attribution 4.0 International license.

**FIG 2:**
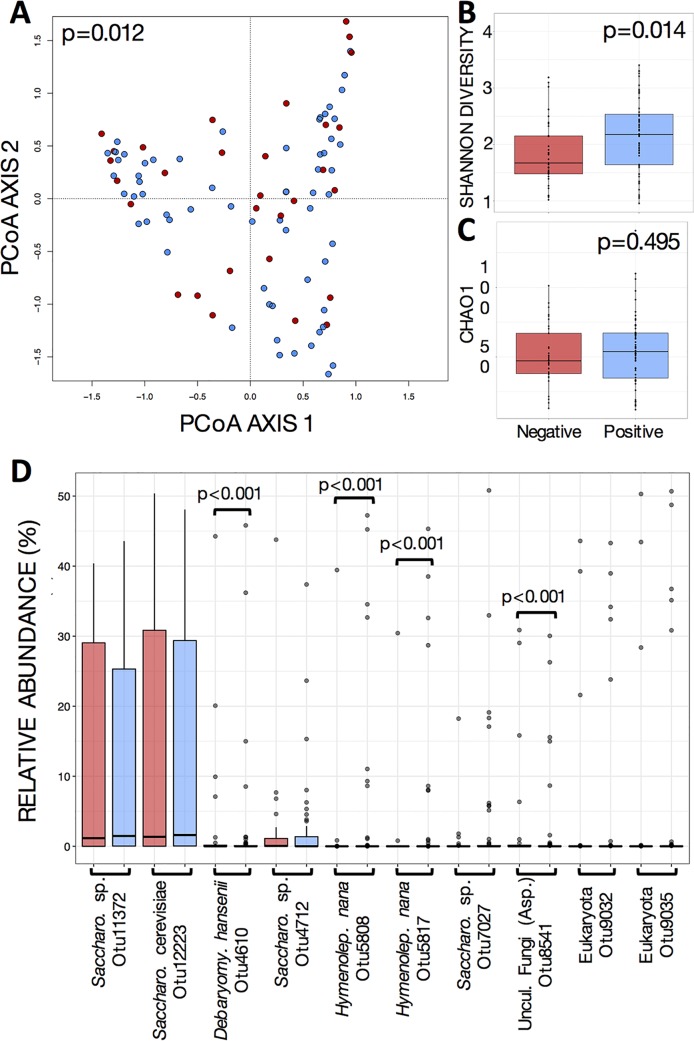
Variations in beta and alpha diversity of gut microbiome eukaryotic communities explained by presence of *Blastocystis*. (A) Principal-component analysis (PCoA) ordination of variation in beta diversity of human gut eukaryotic communities based on Bray-Curtis dissimilarities among samples. Colors represent the presence of *Blastocystis* in gut microbial communities (red for negative and blue for positive). (B and C) Shannon diversity (B) and Chao1 estimated richness (C) display differences in alpha diversity. Significant differences are shown by *P* values of Mann-Whitney tests for comparison between 2 groups. (D) Relative abundances of the 10 most abundant eukaryotic OTUs relative to the presence of *Blastocystis*. Significant differences, calculated by DESeq2 (Wald test plus FDR), are shown.

*Blastocystis* colonization was associated with more discrete differences in the eukaryotic microbiota (Fig. 2). As expected, our analysis detected *Blastocystis* subtype 3 as the protist most significantly different in abundance between *Blastocystis*-positive and -negative individuals (Table 1; see full list in Table S2). *Blastocystis* colonization was also associated with increases in yeast and fungal species (Debaryomyces hansenii, Mucor mucedo, Aspergillus flavus, Mucor racemosus, and Issatchenkia terricola) and a decrease of Hymenolepis nana (Table 1; Table S2). Since H. nana is a known cestode colonizer in the human gut, often with pathological consequences, we evaluated whether the presence of H. nana influenced bacterial community structure and confounded our results. However, we found that H. nana did not drive a significant effect in bacterial community structure (Fig. S2A). The detection of a significant difference in H. nana abundances between *Blastocystis*-positive and -negative individuals is likely due to a high variation in abundance in both groups and relatively few samples where they are represented. Likewise, we investigated the impact of the presence of Debaryomyces hansenii in the microbiota because this yeast species has been associated with human infections (37). Interestingly, the presence of this yeast explained 3.3% of bacterial community variation (PERMANOVA, *P* > 0.001). However, the distribution of D. hansenni did not overlap the distribution of *Blastocystis* (Fig. S2B), and their effects on the bacterial community are independent from each other (PERMANOVA, *P* = 0.68).

10.1128/mSystems.00007-18.2 FIG S2  Influence of Hymenolepis nana and Debaryomyces hansenii on bacterial community structure. Principal-component analysis (PCoA) ordination of variations in beta diversity of human gut bacterial communities based on Bray-Curtis dissimilarities among samples. (A) Colors represent the presence of *Blastocystis* in gut microbial communities (red for negative and blue for positive). Circles highlighted in yellow represent samples positive for Hymenolepis nana. (B) Ordination of bacterial community structures showing samples positive (yellow) or negative (black) for fungus D. hansenii and also showing the presence (triangles) and absence (circles) of *Blastocystis*. Download FIG S2, TIF file, 2.5 MB. Copyright © 2018 Nieves-Ramírez et al. 2018 Nieves-Ramírez et al. This content is distributed under the terms of the Creative Commons Attribution 4.0 International license.

While controlling for sequencing depth, we observed that it interacted significantly with the effect of *Blastocystis* species colonization on community structure (PERMANOVA, *P* = 0.02) (Fig. S3B). This result is biologically intuitive since the presence of *Blastocystis* species in itself is contributing to the number of sequences detected. However, because of this result, we carried out the DESeq2 analysis using variance-stabilizing-transformed data (38). This analysis yielded the same numbers and identities of differential OTUs between *Blastocystis*-colonized and -noncolonized individuals as we obtained with nontransformed data.

10.1128/mSystems.00007-18.3 FIG S3  Influence of sequencing depth on bacterial and eukaryotic community structures. (A and B) Ordination of the bacterial (A) and eukaryotic (B) community structures showing samples positive (triangles) and negative (circles) for *Blastocystis* and also showing sequencing depth using a color scale (increasing sequence numbers from blue to yellow). Download FIG S3, TIF file, 2.4 MB. Copyright © 2018 Nieves-Ramírez et al. 2018 Nieves-Ramírez et al. This content is distributed under the terms of the Creative Commons Attribution 4.0 International license.

Altogether, the eukaryomes’ compositional differences in relation to *Blastocystis* colonization resulted in only a marginal difference in eukaryome beta diversity (PCoA, PERMANOVA, *P* = 0.01) (Fig. 2A). *Blastocystis* colonization was associated with a smaller, yet significant increase in eukaryome alpha diversity (Shannon index, Mann-Whitney, *P* = 0.014) (Fig. 2B) compared to the changes in bacterial diversity. There were no changes in community richness (Chao1, Mann-Whitney, *P* = 0.49).

### (iii) Interdomain associations.

**FIG 3:**
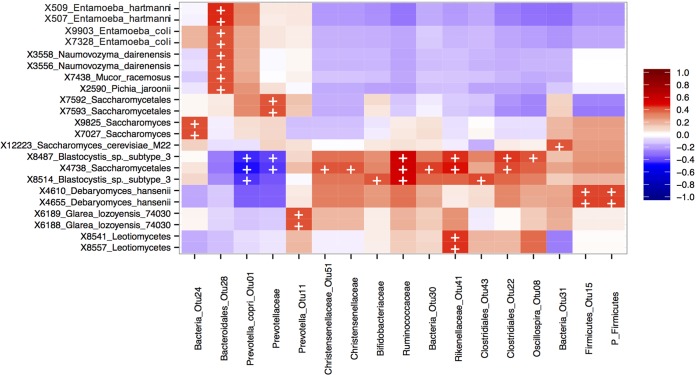
Heatmap of biweight correlations (bicor method) between top 100 bacterial (*x* axis) and top 100 taxon (*y* axis) OTUs in fecal samples from study participants. Colors denote positive (red) and negative (blue) correlation values. Significant correlations are denoted with a plus sign (*P* < 0.05; FDR).

Correlative analysis of the abundances of the top 100 most abundant taxa of the 16S versus 18S rRNA gene data sets showed, as expected, that *Blastocystis* subtype 3 was positively correlated with members of the *Ruminoccaceae* family and inversely correlated with Prevotella copri (Fig. 3).

### Bacterial-community-derived functional changes associated with *Blastocystis* colonization.

**FIG 4:**
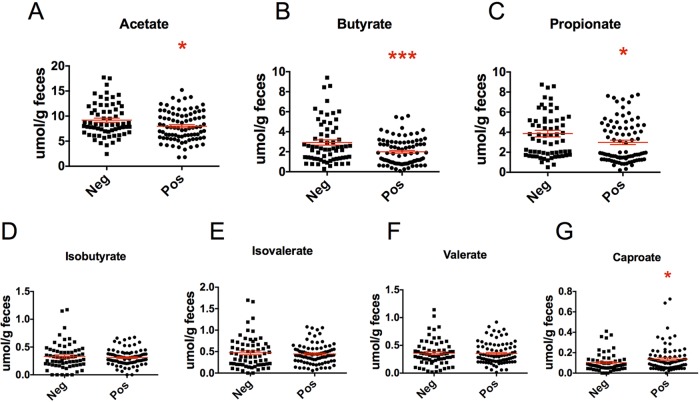
Short-chain fatty acid production in relation to *Blastocystis* colonization. Concentrations of fecal acetate, propionate, butyrate, isobutyrate, isovalerate, valerate, and caproate were measured by gas chromatography (*N*_pos_ = 102, *N*_neg_ = 54; *, *P* < 0.05; ***, *P* < 0.001 [Mann-Whitney]).

To determine the functional differences associated with *Blastocystis* colonization, we inferred metagenomics potential using PICRUSt (39). Metagenomes predicted from the 16S rRNA gene data revealed 202 differential biochemical pathways out of 266 biochemical pathways associated with *Blastocystis* colonization (Welch’s *t* test) (Table S3), strongly suggesting that the microbiomes in the two groups were functionally distinct. The functional differences involved diverse metabolic functions, including metabolism of secondary bile acids, lipids, tryptophan and other amino acids, and carbohydrates. To confirm the predicted changes in carbohydrate metabolism, we next directly measured the concentration of fecal short-chain fatty acids (SCFA) in all samples. As predicted by our PICRUSt analysis, colonization with *Blastocystis* was associated with vastly different bacterial colonic fermentation patterns, as measured by production of SCFA. The three main SCFA, acetate, butyrate, and propionate, were reduced in colonized individuals, whereas caproate was increased (Fig. 4).

### Immunological differences in *Blastocystis*-colonized individuals. (i) Fecal calprotectin.

**FIG 5:**
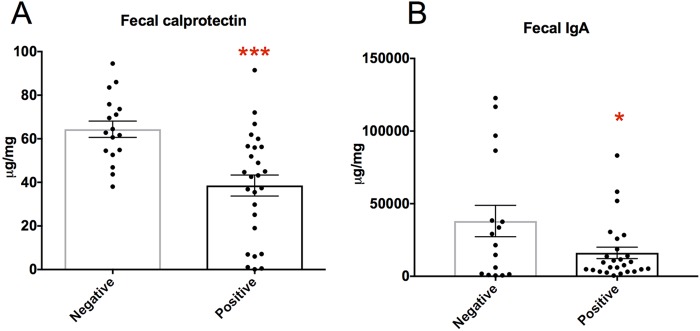
Gut mucosal immune changes associated with *Blastocystis* colonization. Concentrations of calprotectin (A) and total IgA (B) were determined by ELISA in feces in a subset of samples from this study (*N*_neg_ = 17, *N*_pos_ = 26; *, *P* < 0.05; ***, *P* < 0.001 [Mann-Whitney]).

To determine whether *Blastocystis* colonization was not associated with intestinal inflammation (as inferred by the Rome III criteria questionnaires), we measured fecal calprotectin in a subset of samples (*N*_pos_ = 26 and *N*_neg_ = 17). Calprotectin is a neutrophil cytosolic protein commonly used as a marker of gut inflammation (40). Strikingly, lower concentrations of fecal calprotectin were observed in Blastocystis*-colonized individuals than in noncolonized individuals (Mann-Whitney, *P* = 0.0003) (Fig. 5A). All calprotectin values were well under levels consistent with pathological gut inflammation (>200 µg/mg), in accordance with the lack of clinical symptoms noted in our study cohort.

### (ii) Fecal IgA.

Immunoglobulin A (IgA) is the most abundant antibody in mucosal surfaces, and it has a critical role in maintaining homeostasis with the microbiome (41). It acts by binding to and neutralizing invading pathogens, and it can specially target microbes close to the mucus layer (42). IgA secretion into the lumen is elicited by parasitic infections with helminths, and it is important in limiting parasite fecundity and providing immune protection against reinfection (43).

We measured the total IgA concentration in feces to determine if asymptomatic *Blastocystis* colonization was associated with changes in this important humoral immune factor. In a subset of individuals (*N*_pos_ = 26 and *N*_neg_ = 17), we observed that, similar to the calprotectin finding, colonized individuals exhibited lower levels of fecal IgA (Mann-Whitney, *P* = 0.03) (Fig. 5B).

### (iii) Oxidative stress markers in urine.

It has been described that oxidative stress and the presence of free-radical activities in hosts colonized by parasites correlate with the production of specific metabolites, such as advanced oxidative protein product (AOPP) and malondialdehyde (MDA) (44–46). Previous studies have evaluated MDA levels among healthy individuals and people infected by Ascaris lumbricoides (45), Entamoeba histolytica, and Plasmodium vivax (44), and all revealed increased levels of MDA in the infected individuals, suggesting that oxidative stress acts as a mediator of tissue damage concurrent with different parasites. We measured these biomarkers in urine samples of most study participants (*N*_pos_ = 102 and *N*_neg_ = 53), as described previously (13, 47), and found no significant differences between colonized and noncolonized individuals (Table S6), suggesting that *Blastocystis* colonization is not accompanied by oxidative stress in this population.

10.1128/mSystems.00007-18.9 TABLE S6  Oxidative stress biomarkers measured in urine. Download TABLE S6, DOCX file, 0.1 MB. Copyright © 2018 Nieves-Ramírez et al. 2018 Nieves-Ramírez et al. This content is distributed under the terms of the Creative Commons Attribution 4.0 International license.

### (iv) Serum cytokines.

*Blastocystis* ST7 has been shown to induce the expression of proinflammatory cytokines in mouse intestinal explants (15) and in serum of patients with IBS (16). However, in a reduced subset of our population, no differences in the serum concentrations of cytokines interleukin-2 (IL-2), IL-4, IL-6, IL-10, IL-17, tumor necrosis factor alpha (TNF-α), and gamma interferon (IFN-γ) were detected between *Blastocystis*-infected and noninfected individuals (*N*_pos_ = 16 and * N*_neg_ = 9, Mann-Whitney) (Table S7), further supporting the finding that *Blastocystis* colonization in our cohort is not associated with inflammation. This may reflect on the different immune effects exerted by different *Blastocystis* subtypes; previous work reported differential induction of IL-10 but not of IL-12 or IL-8 by subtype (17). However, *Blastocystis* pathogenicity does not directly correlate with subtype identity (4, 10), and pathogenicity is likely a better predictor of cytokine induction. These results reflect data from a very reduced number of blood samples available in this study and should be confirmed in a larger sample size.

10.1128/mSystems.00007-18.10 TABLE S7  Cytokine concentrations in serum samples for a subset of study participants, measured by cytometric bead array. Download TABLE S7, DOCX file, 0.1 MB. Copyright © 2018 Nieves-Ramírez et al. 2018 Nieves-Ramírez et al. This content is distributed under the terms of the Creative Commons Attribution 4.0 International license.

## DISCUSSION

The intestinal microbiota is highly variable among individual humans, and its diversity is affected by factors like diet, sociogeographic setting, antibiotic use, disease, age, and to a lesser degree, genetics (24, 27, 48). A direct association of intestinal parasites, such as helminths and protozoa, with human intestinal microbiota composition and diversity has been previously reported (33, 49). However, the influence of gastrointestinal inflammation had not been previously accounted for, and thus, it remained unanswered whether the microbiome differences associated with parasite colonization were linked to ecological parameters and/or were a direct result of gut inflammation. Our study focused on residents from a rural Mexican community that were confirmed to be healthy and without clinical symptoms. This population provided an ideal cohort to study how the most common human protist altered the ecology of the bacterial and eukaryotic microbiome in the absence of inflammation. Similar to previous studies (2, 34), our results confirm that colonization with *Blastocystis* is strongly associated with broad shifts in the gut-resident bacterial community and an increase in bacterial alpha diversity, providing very strong evidence that the presence of this protist may influence human gut microbial ecology through mechanisms that remain unknown.

In this population, *Blastocystis* colonization was very strongly correlated with a decrease in Prevotella copri and an increase in Ruminococcus bromii, the first and second most abundant bacterial phylotypes in this data set, respectively. These taxa are so commonly observed within the human gut microbiome that individuals are often described as either *Prevotella* rich or *Ruminococcus* rich (50). *Prevotella* species are dominant colonizers of the human gut in nonwesternized populations that consume a plant-rich diet (24, 51), whereas Ruminococcus bromii has been proposed to be a keystone species of the human gut, due to its colonization capacity and exceptional ability to degrade resistant starches (52, 53). In this context, this study reveals a potential role of *Blastocystis* as a discriminant feature between two fundamental bacterial species of the human gut, as Prevotella copri is positively correlated with *Blastocystis* subtype 3 (ST3), while Ruminococcus bromii is negatively correlated with *Blastocystis* ST3. *Blastocystis* colonization is thus a potentially important factor in structuring the gut microbiota architecture.

While other variables could explain these striking differences in microbial communities, age, family relationships, and health status were controlled for in this study. Although a full dietary assessment was not carried out in this population, dietary patterns in this rural population have historically been observed to be highly homogenous, characterized by a high intake of both plant-based sources (corn, legumes, chilies, tomatoes, and other fruits and vegetables) and meat (mainly chicken and beef). Thus, we do not expect our results to be explained by dietary differences among individuals in our study.

Instead, we postulate that *Blastocystis* may exert a predatory effect on bacteria of highly abundant taxa, such as Prevotella copri, which accounts for ~50% of the bacterial abundance in *Blastocystis*-negative individuals. *Blastocystis* is known to graze on bacteria. Dunn et al. (54) reported that the ameboid form of the protist was capable of bacterial engulfment, a process that has been suggested to serve the nutritional needs of encystation (55). Additional evidence of the grazing of *Blastocystis* on bacteria includes (i) the low frequency of the ameboid form in axenic cultures (56) and (ii) the contact between *Blastocystis* pseudopodia and bacteria (57). Bacterial predation is also known to occur in the case of a group of free-living amoebas, which are the only group of organisms that can cause a decrease of a bacterial population from 10^8^ down to 10^5^ per gram of soil (58, 59). Bacterial engulfment has also been described during colonization by the intestinal protist Entamoeba histolytica (60). Interestingly, a previous study showed that the presence of *Entamoeba* (likely including E. histolytica and E. dispar) was associated with an effect on *Prevotellaceae* and* Ruminococcaceae* that was similar to what we found in this study (33).

In the absence of *Blastocystis*, a strong bacterial competitor like Prevotella copri may dominate the community, which limits species richness and community evenness, as was observed in this study (Fig. 1). When present, *Blastocystis* predation on the most abundant bacterial taxa (also known as density-dependent predation) may lower the competition for nutrients and space, which may lead to an increase in bacterial richness and community evenness, as has been shown in this and previous studies (2, 61). This is well supported by food web theory and examples from macro- and microecology (62, 63), where the increase in community diversity through grazing or predation occurs through a top-down control on the strongest competitors, which consequently allows for the colonization and persistence of weaker competitors in the community. Our results do not eliminate the possibility of a bottom-up control, where specific bacterial community structures may favor colonization of the intestine by *Blastocystis*. However, this alternative explanation, where a more diverse community could favor the entry of a predator into the ecosystem, is not well supported by ecology theory (64). Future studies considering both bottom-up and top-down mechanisms are necessary to test various scenarios, including a deterministic influence of competition and predation on the gut microbial communities, which are impossible to assess in an observational cross-sectional study like this one.

Other potential indirect mechanisms for *Blastocystis* to affect the abundance and diversity of the bacterial microbiome are via direct interaction with the intestinal epithelium and the underlying immune tissue. Indeed, our study revealed differences in calprotectin and IgA. IgA-mediated modulation of the bacterial microbiome has been reported in mice lacking IgA (65, 66) or when comparing low-IgA mice to high-IgA mice (67). However, the result observed for IgA in this study was subtle and unlikely to cause such drastic changes in microbiome composition and function. Likewise, the levels detected for fecal calprotectin (a granulocyte marker protein produced at high levels in the cytosol of neutrophils and a well-known marker of intestinal inflammation [40]) are well below levels consistent with clinical inflammation (68), and *Blastocystis* was associated with lower levels. Still, indirect mechanisms need to be ruled out experimentally before concluding that they are not at play in the results obtained in this study.

These subclinical immune differences may also be a result of (i) the large differences in bacterial community structures observed in relation to *Blastocystis* colonization and/or (ii) a direct anti-inflammatory effect of *Blastocystis* on the intestinal mucosa. Evidence of the former mechanism clearly exists. Changes in IgA production have been observed in cohousing and fecal transfer experiments carried out in mice (67), where high-IgA mice that adopted the microbiota from low-IgA mice experienced a decrease in IgA levels, demonstrating the IgA modulatory capacity of the bacterial microbiome (67). There is also evidence of direct effects of *Blastocystis* on the immune system. *Blastocystis* is associated with reduced neutrophil counts in blood (69) and is known to produce serine proteases that degrade secretory IgA (sIgA) (70). These mechanisms must be further studied to understand if *Blastocystis* creates an anti-inflammatory environment in the intestine and if this may explain the asymptomatic status in this population. Ideally, this could be studied in a longitudinal cohort study that includes participants that transition from a noncolonized to a colonized status.

The large taxonomic differences observed were, unsurprisingly, accompanied by broad functional differences, inferred computationally by PICRUSt and measured directly through the determination of fecal SCFA concentration. Interestingly, the difference in the bacterial microbiome associated with *Blastocystis* colonization resulted in an overall decrease in carbohydrate fermentative metabolism. We attribute this to (i) the decrease in abundance of Prevotella copri, an efficient fiber degrader and acetate producer (71), and (ii) the reduced cross-feeding by other bacterial taxa due to the decrease in Prevotella copri. Cross-feeding, whereby a species produces a metabolite that can be used by other species, is a common feature in many microbial communities (72). In the mammalian gut, fiber is degraded into hexoses and pentoses, which are subsequently used as substrates in pathways that lead to the production of acetate, propionate, and butyrate (71). Butyrate can also be produced from acetate (via acetyl coenzyme A [acetyl-CoA]) (71), which could explain the larger decrease in butyrate concentrations in *Blastocystis*-colonized individuals.

Our study found similarities with previous reports. For example, Audebert et al. also reported increases in bacterial alpha diversity and in the abundance of *Ruminococcus* species in association with *Blastocystis* colonization (2). However, our study found taxonomic discrepancies with previous reports. Previously, O’Brien Andersen et al. (34) had reported an increase in *Prevotella* species associated with *Blastocystis* in a cohort of Danish patients referred to their study by doctors. Likewise, Audebert et al. (2) reported a positive association between *Prevotellaceae* and *Blastocystis* colonization in French individuals. These discrepancies may arise from comparing westernized populations, expected to have different microbiomes than rural Mexicans. Members of the *Prevotellaceae* family account for ~30% of the total gut bacterial taxa in this study, compared to ~5% of the French population studied previously (2). A study that compared the microbiota in relation to *Blastocystis* using shotgun metagenomics data from 11 studies across four continents found an association between *Blastocysis*, Prevotella copri, and Ruminococcus gnavus that was the opposite of what we observed in this study (7) and did not detect a difference in bacterial alpha diversity. These differences may be due to comparing our results to studies that have analyzed a combination of healthy and diseased individuals or because of differences in *Blastocystis* subtypes (2, 34). For example, while colonization was mostly explained by *Blastocystis* subtype 3 in this study, the samples analyzed by Audebert et al. included colonization with many more subtypes, including ST1, -2, -3, -4, -6, and -7 (2). Notably, an ongoing study in asymptomatic individuals from Cameroon has revealed findings similar to those of our study in relation to *Blastocystis* colonization (Laure Segurel, personal communication), suggesting that the effect of *Blastocystis* on the bacterial microbiome may be dependent on the sociogeographical setting studied or on similarities in subtype colonization. The important interstudy discrepancies reflect the need to study *Blastocystis* in a context-specific manner, accounting for the effects of subtypes, inflammation, geographical setting, and other covariates that may influence microbiome composition and diversity.

Altogether, our work is the first to show the important ecological association between the most common human-associated protist and the fundamental species of the bacterial microbiome in the absence of gastrointestinal disease or inflammation. While our findings are in agreement with the well-supported top-down effect of a predator on ecosystem diversity, this remains to be tested experimentally. If proven, the presence of *Blastocystis* or similar bacterial predators may be an important mechanism of microbial diversity maintenance that is not currently being considered in the human microbiome field. Including microbial eukaryotes in human gut microbiome surveys will provide a more integrated understanding of the ecological processes that shape the gut microbiome and the mechanisms by which they relate to health and disease.

## MATERIALS AND METHODS

### Study population and study design.

This cross-sectional study was carried out in Xoxocotla, Morelos, Mexico, about 120 km south of Mexico City (longitude 99°19′W, latitude 18°3′N). This semirural area spans 29,917 km^2^ and hosts a total population of 5,163 people. The region is characterized by tropical climate (warm subhumid), and agriculture is the main source of economic income. Sample collection was carried out between January and September 2014. A total of 156 urine and stool samples were collected from individuals at Xoxocotla Health Center as a part of a routine medical examination of people living in the town or within the vicinity. Urine and stool samples were collected in sterile plastic containers, immediately placed at 4°C for transport, and then stored at −20°C until analysis. Prior to sample and data collection, the nature of the study was explained to the participants and informed verbal and written consents were obtained. Demographic, socioeconomic, and environmental data and the history of gastrointestinal symptoms in the 3 months preceding sampling (abdominal pain or discomfort and defecation patterns) were obtained based on the standardized Rome III questionnaire in face-to-face interviews. The Rome classification system is based on the symptom clusters that remain consistent across clinical and population groups and is currently the gold standard to determine gut health (73). The protocol was approved by the Medical Ethics Committee of the National Autonomous University of Mexico.

### Real-time qPCR analysis for detection of *Blastocystis* and other parasites.

The presence of *Blastocystis*, Cryptosporidium parvum, Entamoeba histolytica/E. dispar, and Giardia intestinalis was assessed by quantitative PCR (qPCR) using QuantiTect SYBR green master mix (Qiagen). The *Blastocystis*-specific primer BhRDr (5′ GAG CTT TTT AAC TGC AAC AAC G 3′ [74]) and the broad-specificity eukaryote-specific primer RD5 (5′ ATC TGG TTG ATC CTG CCA GT 3′ [75]), the *Cryptosporidium*-specific primers CrF (forward [F], 5′ CGC TTC TCT AGC CTT TCA TGA 3′) and CrR (reverse [R], 5′ CTT CAC GTG TGT TTG CCA AT 3′), the Entamoeba histolytica/E. dispar-specific primers Ehd-239F (5′ ATT GTC GTG GCA TCC TAA CTC A 3′) and Ehd-88R (5′ GCG GAC GGC TCA TTA TAA CA 3′), and the *Giardia*-specific primers Giardia-80F (F, 5′ GAC GGC TCA GGA CAA CGG TT 3′) and *Giardia*-127R (R, 5′ TTG CCA GCG GTG TCC G 3′) (76) were used. Amplification reactions were performed in 10-µl reaction mixture volumes with the *Taq* PCR master mix kit (Qiagen) and 6.25 pmol each of primers BhRDr-RD5, CrF-CrR, Ehd-239F–Ehd-88R, and *Giardia*-80F–*Giardia*-127R. The amplification conditions consisted of 35 cycles of 1 min each at 94, 59, and 72°C, with an additional step of 95°C for 15 s, 60°C for 1 min, 95°C for 30 s, and 60°C for 15 s (77). The qPCR plates included positive controls (samples known to be positive for each parasite), as well as standard curves using DNA from each parasite from ATCC’s enteric protozoon DNA panel.

The 18S rRNA gene was amplified using primers 5′ GTA CAC ACC GCC CGT C 3′ (F) and 5′ TGA TCC TTC TGC AGG TTC ACC TAC 3′ (R). The amplification conditions consisted of 35 cycles of 1 min each at 94, 59, and 72°C, with an additional step consisting of 95°C for 15 s, 60°C for 1 min, 95°C for 30 s, and 60°C for 15 s. All qPCRs were performed on an Applied Biosystems 7500 machine. The parasitic loads in the samples were calculated as the difference between the average cycle threshold (*C*_*T*_) value of each parasite and the average *C*_*T*_ value of the 18S rRNA gene of each sample.

### Fecal microbial community analysis. (i) Extraction.

DNA was extracted from ~50 mg of stool. Samples were mechanically lysed using Mo Bio dry bead tubes (Mo Bio Laboratories, Inc.) and the FastPrep homogenizer (FastPrep instrument; MP Biochemicals) before DNA extraction with the Qiagen DNA stool minikit.

### (ii) Amplification.

For 16S rRNA gene amplification, samples were amplified by PCR in triplicate using bar-coded primer pairs flanking the V3 region of the 16S rRNA gene as previously described (78). Each 50 ml of PCR mixture contained 22 ml of water, 25 ml of TopTaq master mix, 0.5 ml of each forward and reverse bar-coded primer, and 2 ml of template DNA. The PCR program consisted of an initial DNA denaturation step at 95°C (5 min), 25 cycles of DNA denaturation at 95°C (1 min), an annealing step at 50°C (1 min), an elongation step at 72°C (1 min), and a final elongation step at 72°C (7 min). Controls without template DNA were included to ensure that no contamination occurred. Amplicons were run on a 2% agarose gel to ensure adequate amplification. Amplicons displaying bands at ~160 bp were purified using the illustra GFX PCR DNA purification kit. Purified samples were diluted 1:50 and quantified using PicoGreen (Invitrogen) in the Tecan M200 plate reader (excitation at 480 nm and emission at 520 nm).

### (iii) Sequencing.

For 16S rRNA gene sequencing, each PCR pool was analyzed on the Agilent Bioanalyzer using the high-sensitivity double-stranded DNA (dsDNA) assay to determine approximate library fragment size and verify library integrity. Pooled-library concentrations were determined using the TruSeq DNA sample preparation kit, version 2 (Illumina). Library pools were diluted to 4 nM and denatured into single strands using fresh 0.2 N NaOH. The final library loading concentration was 8 pM, with an additional PhiX spike-in of 20%. Sequencing was carried out using a Hi-Seq 2000 bidirectional Illumina sequencing and cluster kit, version 4 (Macrogen, Inc.). The 18S rRNA gene was sent to the Integrated Microbiome Resource at Dalhousie University for amplification and sequencing. The 18S rRNA gene was amplified with the primers E572F (5′ CYGCGGTAATTCCAGCTC 3′) and E1009R (5′ AYGGTATCTRATCRTCTTYG 3′), and the reaction mixture included a PNA blocking primer (5′ TCTTAATCATGGCCTCAGTT 3′) to reduce amplification of mammalian sequences. Amplification was carried out in duplicate, with one reaction mixture using undiluted DNA and the other using DNA diluted 1:10 in PCR water. Amplification was conducted according to previously described protocols (79). PCR products were visualized on E-gels, quantified using Invitrogen Qubit with PicoGreen, and pooled at equal concentrations, according to a previous report (79). PhiX was spiked in at 5%, and the resulting library was sequenced at Dalhousie University on the Illumina MiSeq using the MiSeq 500-cycle reagent kit, version 2 (250 × 2).

### Bioinformatics.

Sequences were preprocessed, denoised, and quality filtered by size using the Mothur MiSeq SOP (standard operation protocol) (16S rRNA gene [80]) or QIIME (18S rRNA gene [81]).

### (i) 16S rRNA gene sequences.

All sequences were processed using Mothur according to the standard operating procedure as previously described (82). Quality sequences were obtained by removing sequences with ambiguous bases, a low-quality read length, and/or chimeras identified using chimera.uchime. Quality sequences were aligned to the SILVA bacterial reference alignment, and OTUs were generated using a dissimilarity cutoff of 0.03. Sequences were classified using the classify.seqs command.

### (ii) 18S rRNA gene sequences.

Demultiplexed reads were trimmed to a uniform length of 250 bp using the FastX-Toolkit (http://hannonlab.cshl.edu/fastx_toolkit/) and clustered into operational taxonomic units (OTUs) using the minimum entropy decomposition (MED) method (83) as implemented in the oligotyping microbial analysis software package (84). MED performs *de novo* taxonomic clustering using Shannon entropy to separate biologically meaningful patterns of nucleotide diversity from sequencing noise; the processed data are partitioned into phylogenetically homogeneous units (MED nodes) for downstream bacterial diversity analyses. This analysis was carried out with the minimum substantive abundance parameter (−M) set at 250 reads. All other parameters were run with default settings; the maximum variation allowed per node (−V) was automatically set at 3 nucleotides.

Representative sequences were classified by clustering against the Greengenes Database at 97% similarity (16S rRNA gene [85]) or SILVA release 123 at 99% similarity (18S rRNA gene [86]). The 16S rRNA gene data set was filtered to remove mitochondrion and chloroplast sequences and OTUs present in fewer than three samples. The 18S rRNA gene data set was filtered to remove mammalian and plant sequences and all OTUs present in fewer than three samples. Both data sets were filtered to exclude singletons and doubletons. Following filtering, a cutoff of 1,000 reads per sample was applied. All 16S rRNA gene samples passed the cutoff, while 63 samples were excluded from the 18S rRNA gene data set.

### PICRUSt.

We used PICRUSt (39) to generate a profile of putative functions (via metagenome prediction) from the 16S rRNA OTU data. Predicted metagenomes from all the samples were categorized by function at KEGG Orthology level 3. To test for significant differences in functional category abundances between colonized and noncolonized individuals, we used the Welch’s *t* test implementation of STAMP (87).

### SCFA analysis.

Fecal samples were combined with 25% phosphoric acid, vortexed, and centrifuged until a clear supernatant was obtained. Supernatants were submitted for GC analysis to the Department of Agricultural, Food and Nutritional Science of the University of Alberta. Samples were analyzed as previously described (88), with modifications. Briefly, samples were combined with 4-methyl-valeric acid as an internal standard, and 0.2 ml was injected into the Bruker Scion 456 gas chromatograph, using a Stabilwax-DA 30-m by 0.53-mm by 0.5-µm column (Restek). A standard solution containing acetic acid, propionic acid, isobutyric acid, butyric acid, isovaleric acid, valeric acid, and caproic acid combined with internal standard was injected in every run.

The PTV (programmable temperature vaporization) injector and FID (flame ionization detector) detector temperatures were held at 250°C for the entire run. The oven was started at 80°C and immediately ramped to 210°C at 45°C/min, where it was held for 5.11 min. Total run time was 8.00 min. Helium was used at a constant flow of 20.00 ml/min. Sample concentrations were normalized to the wet weight of feces.

### Calprotectin assay.

Fecal calprotectin was determined using a human calprotectin enzyme-linked immunosorbent assay (ELISA) kit (Hycult Biotech, Inc., Uden, Netherlands). Stool extracts were prepared and analyzed according to the manufacturer’s instructions. After normalizing fecal weight to 1-mg/µl solutions, samples were diluted 10× in phosphate-buffered saline (PBS). The standard curve ranged between 1.6 and 50 ng/ml.

### IgA assay.

Fecal IgA concentrations were measured by enzyme-linked immunosorbent assay (Chondrex) in samples diluted 1:1,000. After normalizing fecal weight to 1-mg/µl solutions, samples were diluted 1,000× in PBS. The standard curve ranged between 1.6 and 50 ng/ml.

### Oxidative stress biomarkers in urine.

Biochemical assays for AOPP and MDA were carried out on collected urine specimens according to methods previously established (13, 47). AOPP was measured spectrophotometrically according to a previously described method (89), using a microplate reader (Tecan Infinite M200; Tecan, Switzerland) with the following modifications. Briefly, 200-µl amounts of urine diluted 1:5 in PBS or chloramine-T standard solutions (0 to 100 µmol/liter) were mixed with 20 µl of acetic acid and 10 µl of 1.16 M potassium iodide (KI; Sigma). The absorbance was immediately read at 340 nm against a blank containing 200 µl of PBS, 20 µl of acetic acid, and 10 µl of KI. AOPP concentrations were expressed in µmol/liter of chloramine-T equivalents.

The free radical-induced lipid peroxidation level in urine was determined by measuring MDA with an assay modified from the thiobarbituric acid-reactive-substance method (90). Briefly, 600-µl amounts of urine or 1,1,3,3-tetraethoxypropane standard solutions (0.15, 0.30, 0.60, 1.20, 2.40, 5.0, and 10.0 nmol/ml) were mixed with 300 µl of 37% hydrochloric acid. After centrifugation (800 × *g* for 10 min), 500 µl of 0.65% *N*-methyl-2-phenylindole (MPI) (acetonitrile-methanol [3:1] diluent; Sigma) and 500 µl of deproteinized serum were added, followed by a 45-min incubation at 45°C. The resulting blue-colored chromophore was measured at an absorbance of 586 nm in a microplate reader (Tecan Infinite M200; Tecan, Switzerland) using 1,1,3,3-tetraethoxypropane as a standard and water as the blank. Concentrations were expressed as nmol/ml.

### Serum IL measurement.

Serum was extracted from 5 ml of peripheral blood of each individual to measure IL-2, IL-4, IL-6, IL-10, TNF, IFN-γ, and IL-17A protein levels according to the manufacturer’s recommendations for the BD cytometric bead array (CBA) human Th1/Th2/Th17 cytokine kit (BD Biosciences, San Jose CA, USA).

### Statistical analysis.

Differences in frequencies for categorical and continuous variables between cases and controls were evaluated using the chi-squared and Student’s *t* test, respectively. We assessed fecal microbial diversity and the relative abundances of bacterial and eukaryotic taxa using phyloseq (91), along with additional R-based computational tools (92–97). PCoAs were conducted using phyloseq (Bray-Curtis dissimilarities as distance metric) on both variance-stabilizing-transformed and rarefied OTU matrices and then statistically confirmed by a permutational multivariate analysis of variance (PERMANOVA) to confirm our results were not a consequence of heteroscedastic dispersion between groups (38). The Shannon and Chao1 alpha diversity indices were calculated using phyloseq and statistically confirmed by the Mann-Whitney test (GraphPad Prism software, version 5c). The R packages DESeq2 (98) and MaAsLin (35) were used to calculate differentially abundant OTUs. Correlation analysis was performed using the bicor method in the R package microbiome to correlate the 100 most abundant OTUs from the 16S and 18S rRNA gene data sets. Features in the analysis were included as OTUs and as OTUs combined into taxonomic families.

To confirm that the effects of *Blastocystis* on the gut bacterial community structure were not due to the presence and interaction of other protozoans or influenced by variation in sequencing depth, we used PERMANOVA models. We built three models, one each for *Blastocystis* with Hymenolepis nana, *Blastocystis* with Debaryomyces hansenii, and *Blastocystis* with sequencing. Neither protozoan interacted significantly with the effect of *Blastocystis* on the gut bacterial community structure, and *Blastocystis* remained a significant driver of community structure in both models. For the 18S rRNA gene data set, a PERMANOVA on gut eukaryotic community structure shows that the sequencing depth appears to interact significantly with the *Blastocystis* species colonization effect on community structure. This result is biologically intuitive, since the presence of *Blastocystis* species in itself is contributing to the number of sequences detected. However, to further control for this result, we carried out the DESeq2 analysis using variance-stabilizing-transformed data. This analysis yielded the same number and identity of differential OTUs between *Blastocystis*-colonized and -noncolonized individuals as we obtained with nontransformed data.

The statistical analyses for calprotectin, IgA, serum cytokines, SCFA, and urine oxidative stress markers were performed with GraphPad Prism software, version 6.00 (GraphPad Software, Inc., San Diego, CA, USA). For comparison between two groups, Student’s *t* tests and Mann-Whitney tests were used for normally and abnormally distributed data sets, respectively, and no samples were excluded from statistical analysis. Statistically significant differences were set at a *P* value of ≤0.05. A *P* value of >0.05 was considered not significant.

### Data availability.

Sequences are publicly available at https://doi.org/10.6084/m9.figshare.6359306. The R code supporting the results of this article is available in the Xoxocotla Gut Microbiome project repository on figshare at https://figshare.com/s/a99310b6d9be4e9de4fe.
