# Supplementary material for: Asymptomatic Intestinal Colonization with Protist Blastocystis Is Strongly Associated with Distinct Microbiome Ecological Patterns
Source: mSystems. 2018 Jun 26;3(3):e00007-18. doi: 10.1128/mSystems.00007-18 (PMC6020473; doi:10.1128/mSystems.00007-18)
Supplement: TABLE S1 [file sys003182239st1.docx]

| **Variable** | **Negative**  **(n=54)** | **Positive**  **(n=102)** | **P value** |
| --- | --- | --- | --- |
| **Gender**  Female  Male | 30 (56%)  24 (44%) | 53 (52%)  49 (48%) | 0.67 |
| **Age (years)** | 28.1±7.9 | 27.8±8.4 | 0.83 |
| **Schooling level**  None  Preschool  Elementary  High School  Post-secondary  No data | 6 (11%)  1 (2%)  20 (37%)  20 (37%)  3 (6%)  4 (7%) | 10 (10%)  0 (0%)  34 (33%)  40 (39%)  15 (15%)  3 (3%) | 0.28 |

Table S1
