# Supplementary figures and images for: Asymptomatic Intestinal Colonization with Protist Blastocystis Is Strongly Associated with Distinct Microbiome Ecological Patterns
Source: mSystems. 2018 Jun 26;3(3):e00007-18. doi: 10.1128/mSystems.00007-18 (PMC6020473; doi:10.1128/mSystems.00007-18)

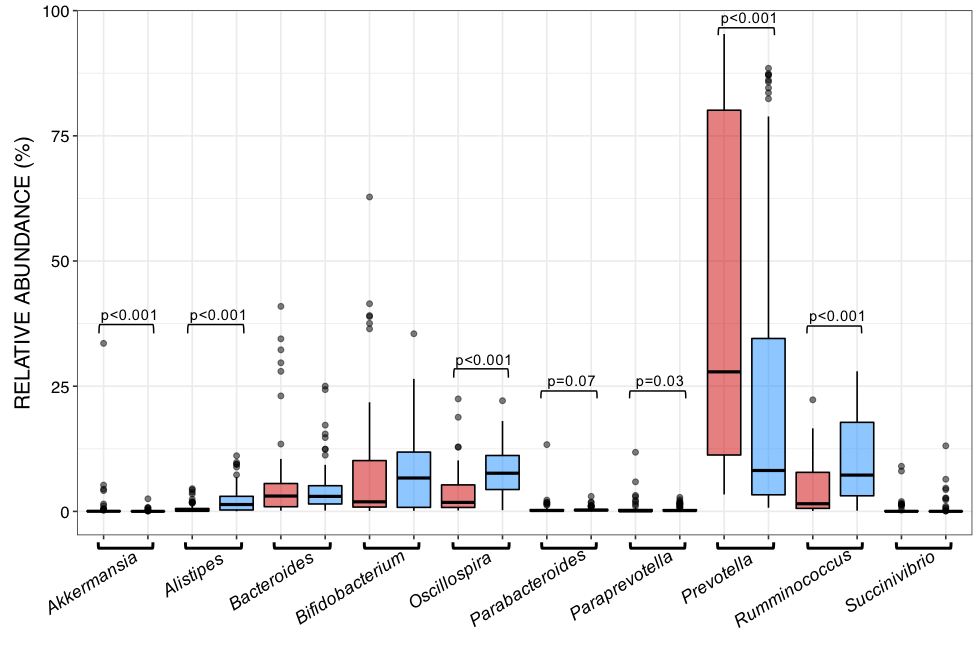

Supplement: FIG S1 [file sys003182239sf1.tif]

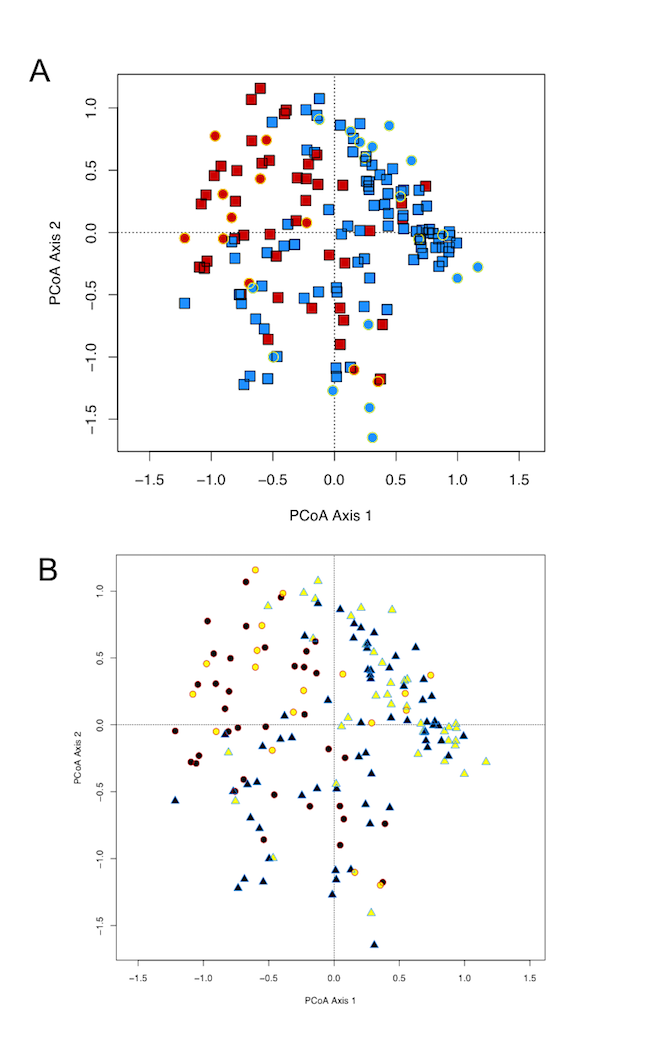

Supplement: FIG S2 [file sys003182239sf2.tif]

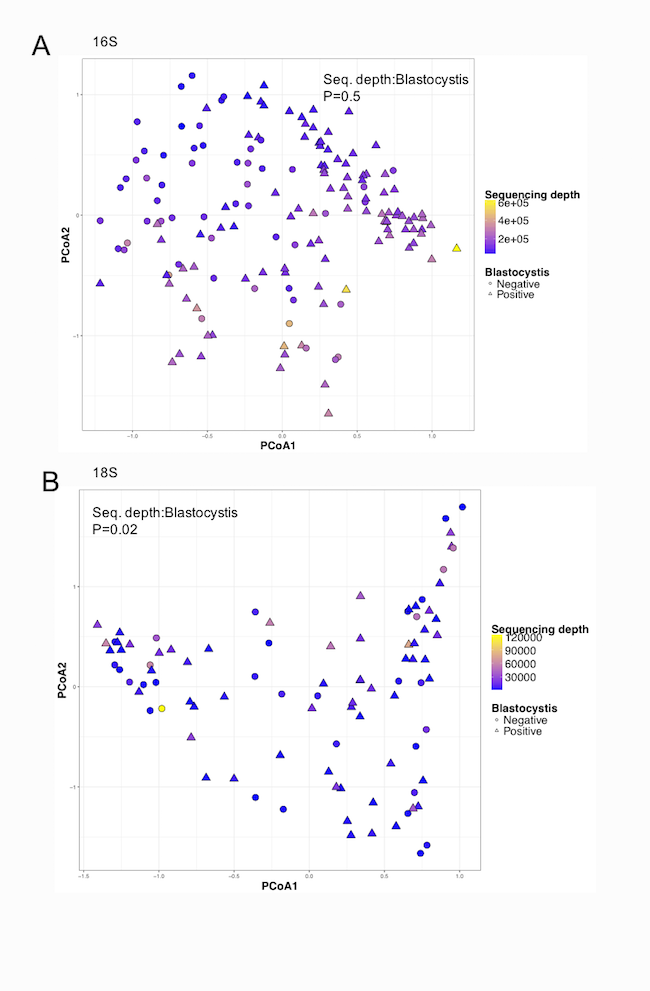

Supplement: FIG S3 [file sys003182239sf3.tif]
