# Supplementary material for: Asymptomatic Intestinal Colonization with Protist Blastocystis Is Strongly Associated with Distinct Microbiome Ecological Patterns
Source: mSystems. 2018 Jun 26;3(3):e00007-18. doi: 10.1128/mSystems.00007-18 (PMC6020473; doi:10.1128/mSystems.00007-18)
Supplement: TABLE S4 [file sys003182239st4.docx]

| Variable | Feature | Coefficient | P.value | Q.value |
| --- | --- | --- | --- | --- |
| Blastocystis | Bacteroides\|Otu00047 | 0.026 | 1.37E-05 | 1.37E-04 |
| Blastocystis | Barnesiellaceae\|Otu00032 | 0.053 | 7.06E-07 | 1.31E-05 |
| Blastocystis | Paraprevotella\|Otu00039 | 0.010 | 5.21E-03 | 2.48E-02 |
| Blastocystis | Prevotella\|copri\|Otu00001 | -0.165 | 2.16E-04 | 1.44E-03 |
| Blastocystis | Prevotella\|copri\|Otu00002 | -0.009 | 8.52E-03 | 3.87E-02 |
| Blastocystis | Prevotella\|copri\|Otu00016 | -0.006 | 1.12E-03 | 6.58E-03 |
| Blastocystis | Alistipes\|putredinis\|Otu00018 | 0.062 | 6.06E-07 | 1.31E-05 |
| Blastocystis | Rikenellaceae\|Otu00034 | 0.017 | 5.58E-04 | 3.49E-03 |
| Blastocystis | Rikenellaceae\|Otu00041 | 0.036 | 9.24E-05 | 7.11E-04 |
| Blastocystis | Lachnospiraceae\|Otu00038 | 0.012 | 1.14E-02 | 4.95E-02 |
| Blastocystis | Clostridiales\|Otu00014 | 0.047 | 9.06E-10 | 9.06E-08 |
| Blastocystis | Clostridiales\|Otu00022 | 0.069 | 2.07E-08 | 1.03E-06 |
| Blastocystis | Clostridiales\|Otu00043 | 0.043 | 7.87E-07 | 1.31E-05 |
| Blastocystis | Oscillospira\|Otu00008 | 0.096 | 4.36E-08 | 1.45E-06 |
| Blastocystis | Oscillospira\|Otu00025 | 0.034 | 1.02E-04 | 7.31E-04 |
| Blastocystis | Ruminococcaceae\|Otu00020 | 0.013 | 2.63E-03 | 1.38E-02 |
| Blastocystis | Ruminococcus\|bromii\|Otu00006 | 0.109 | 1.24E-05 | 1.37E-04 |
| Blastocystis | Bacteria\|Firmicutes\|Otu00015 | 0.058 | 7.71E-06 | 9.64E-05 |
| Blastocystis | Bacteria\|Otu00009 | 0.106 | 2.25E-06 | 3.22E-05 |
| Blastocystis | Bacteria\|Otu00027 | 0.040 | 1.80E-05 | 1.63E-04 |
| Blastocystis | Bacteria\|Otu00030 | 0.039 | 5.51E-05 | 4.59E-04 |
| Blastocystis | Bacteria\|Otu00031 | 0.029 | 3.23E-03 | 1.62E-02 |

Table S4
