# Supplementary material for: Asymptomatic Intestinal Colonization with Protist Blastocystis Is Strongly Associated with Distinct Microbiome Ecological Patterns
Source: mSystems. 2018 Jun 26;3(3):e00007-18. doi: 10.1128/mSystems.00007-18 (PMC6020473; doi:10.1128/mSystems.00007-18)
Supplement: TABLE S5 [file sys003182239st5.docx]

Table S5

| Variable | Negative  (n=33) | Positive  (n=60) | P value |
| --- | --- | --- | --- |
| **Gender**  Female  Male | 20 (61%)  13 (39%) | 32 (53%)  28 (47%) | 0.5 |
| **Age (years)** | 27.2±9.2 | 27.9±7.7 | 0.69 |
| **Schooling level**  None  Preschool  Elementary  High School  Post-secondary  No data | 3 (10%)  0 (0%)  11 (33%)  15 (46%)  2 (5%)  2 (6%) | 6 (10%)  0 (0%)  23 (38%)  24 (40%)  5 (9%)  2 (3%) | 0.92 |
