# Supplementary material for: Asymptomatic Intestinal Colonization with Protist Blastocystis Is Strongly Associated with Distinct Microbiome Ecological Patterns
Source: mSystems. 2018 Jun 26;3(3):e00007-18. doi: 10.1128/mSystems.00007-18 (PMC6020473; doi:10.1128/mSystems.00007-18)
Supplement: TABLE S6 [file sys003182239st6.docx]

Table S6

| **Groups** | **Number of samples** | **AOPP**  **(umol/L)**  Mean ± S.E.M | **MDA**  **(umol/L)**  Mean ± S.E.M |
| --- | --- | --- | --- |
| ***Blastocystis*-negative** | 53 | 157 ± 11 | 0.10 ± 0.0057 |
| ***Blastocystis*-positive** | 102 | 157 ± 12 | 0.10 ± 0.0072 |
| **Student T test**  ***p* value** | - - - | 0.9975 | 0.9027 |
