# Supplementary material for: Asymptomatic Intestinal Colonization with Protist Blastocystis Is Strongly Associated with Distinct Microbiome Ecological Patterns
Source: mSystems. 2018 Jun 26;3(3):e00007-18. doi: 10.1128/mSystems.00007-18 (PMC6020473; doi:10.1128/mSystems.00007-18)
Supplement: TABLE S7 [file sys003182239st7.docx]

Table S7

| **Cytokines** | **Blastocystis-negative**  **(n=9) (pg/mL)**  Mean ± S.E.M | **Blastocystis-positive**  **(n=16) (pg/mL)**  Mean ± S.E.M | **Student t test**  ***p* value** |
| --- | --- | --- | --- |
| **IFN-gamma** | 2.0 ± 1.4 | 0.67 ± 0.38 | 0.2497 |
| **IL-2** | 0.022 ±0.022 | 0.058 ± 0.040 | 0.5370 |
| **IL-4** | 0.071 ± 0.065 | 0.28 ± 0.11 | 0.1677 |
| **IL-6** | 5.6 ±1.7 | 10 ± 5.4 | 0.5496 |
| **IL-10** | 1.2 ± 0.24 | 1.2 ± 0.29 | 0.9119 |
| **IL-17A** | 22 ±15 | 16 ± 5.1 | 0.6396 |
| **TNF** | 0.56 ± 0.23 | 2.5 ± 1.7 | 0.4136 |
